# Pediatric autoimmune neuropsychiatric disorders associated with streptococcal infections and gut microbiota composition: what do we know?

**DOI:** 10.3389/fnut.2024.1477893

**Published:** 2025-01-06

**Authors:** Veronica Maria Tagi, Martina Tosi, Ilenia Pia Greco, Eliana Stucchi, Elvira Verduci, Gianvincenzo Zuccotti

**Affiliations:** ^1^Department of Pediatrics, Vittore Buzzi Children's Hospital, University of Milan, Milan, Italy; ^2^Department of Biomedical and Clinical Science, University of Milan, Milan, Italy; ^3^Department of Health Sciences, University of Milan, Milan, Italy; ^4^Metabolic Diseases Unit, Department of Pediatrics, Vittore Buzzi Children's Hospital, University of Milan, Milan, Italy

**Keywords:** PANDAS, gut microbiota, nutrition, diet, gut-brain axis, oxidative stress

## Abstract

Post-streptococcal autoimmune neuropsychiatric disorders (PANDAS) are a group of pathological condition characterized by sudden-onset obsessive-compulsive and tic disorders following beta-hemolytic Streptococcus group A (GAS) infection, hypothesized to be caused by autoimmune mechanisms targeting the basal ganglia. Scant literature is available regarding the microbiota composition in children with PANDAS, however few studies support the hypothesis that streptococcal infections may alter gut microbiota composition in these patients, leading to chronic inflammation that may impact the brain function and behavior. Notable changes include reduced microbial diversity and shifts in bacterial populations, which affect metabolic functions crucial for neuroinflammation. Elevated serum levels of sNOX2-dp and isoprostanes indicate oxidative stress, while the presence of lipopolysaccharides (LPS) may contribute to neuroinflammation. The aim of this narrative review is to explore the link between PANDAS and gut microbiota composition. The potential connection between gut microbiota and neuropsychiatric symptoms in PANDAS might suggest the importance of dietary interventions, such as promoting the Mediterranean diet and fiber intake, to reduce the inflammatory state of this patients and therefore improve their outcome.

## Introduction

Pediatric Autoimmune Neuropsychiatric Disorders Associated with Streptococcal Infections (PANDAS) represent a group of neurological tardive complications of *Streptococcus pyogenes* (beta-hemolytic Streptococcus group A, GAS) infection in childhood (1). PANDAS were first described as conditions of brain’s neurologic function impairment resulting in the sudden manifestation of obsessive-compulsive disorders (OCD), tic disorders or other behavioral symptoms due to the complications of GAS infection (1, 2). The close relation with streptococcus infection led to the hypothesis of autoimmune pathogenesis of PANDAS. An autoantibody mimicry mechanism may cause progressive damage of basal ganglia, leading to neuropsychiatric behaviors (3). The diagnosis of PANDAS should be made in presence of OCD and/or tics, complex or not observable in other disorders, with an acute onset and severe episodic changes in behavior between the age of 3 years old and puberty, associated with GAS infection confirmed by positive pharyngeal swab and/or increased titers of anti-streptolysin-O (ASLO) or anti-DNase B. PANDAS most frequently occur in male children or adolescents (4, 5). Characteristic symptoms are tics, hyperactivity, urinary urgency, anxiety, depression, impulsiveness, oppositional defiant disorder eating disorders, and a decline in school performance (1). Studies have shown that, after initial infection, disease exacerbations could be associated with other factors than GAS, such as different bacterial or viral infections, or internal stimuli like stress. Pediatric Acute-Onset Neuropsychiatric Syndrome (PANS) refers to the onset of similar symptoms secondary to other bacteria or viral infections (6, 7).

### PANDAS treatment

Treatment of PANDAS mainly includes psychoactive drugs, immunotherapeutic with steroids, antibiotics, plasmapheresis, and intravenous immunoglobins (1). The administration of antibiotics, in particular penicillin, is useful only in case of active streptococcal infection to eradicate the bacteria (1, 6). Regarding tonsillectomy as a treatment, its effectiveness in limiting OCD symptoms is still debated. Further studies are needed to demonstrate clear evidence of benefit (8).

Most frequently used antipsychotic drugs include risperidone, for severe behavioral symptoms, selective serotonin re-uptake inhibitors (SSRIs), for the improvement of OCD symptoms, atomoxetine, used in presence of attention deficit hyperactivity disorder (ADHD), and lorazepam, for the improvement of motor activity and expressive language. Unfortunately, only few studies have inquired into the effects of psychiatric therapies in PANDAS. Additionally, this treatment includes also psychoactive medications and behavioral-cognitive therapy for children who present severe stress and anxiety. Finally, there is scarce evidence concerning PANDAS treatment with immunotherapy (1, 9): this includes therapies with corticosteroids or cyclooxygenase (COX) inhibitors, probiotic treatment, IVIG, and plasma exchange. Corticosteroid or non-steroidal anti-inflammatory drugs have been observed to reduce the duration of symptom flares, while IntraVenous ImmunoGlobulin (IVIG) and plasma exchange have been demonstrated to significantly reduce Yale-Brown Obsessive Compulsive Scale scores (6). Few studies have indicated that therapeutic manipulations of the composition of the gut microbiota might be an additional treatment for some neuropsychiatric symptoms.

### The role of gut microbiota

The term microbiota refers to the composition of commensal microbes (bacteria, viruses, fungi) in the body of a healthy individual (1, 6). This complex system develops during intrauterine periods and is influenced by various factors as maternal antimicrobial treatments, vaccinations, exposure to chemicals, diet, type of delivery, and infant feeding habits. Research has explored the relationship between the gut microbiota and the development of psychiatric disorders (10, 11): in fact, the hypothesis of the microbiota-gut-brain axis could explain the correlation between the development of the central nervous and gastrointestinal homeostasis (12). Their communication occurs through a variety of complex mechanisms involving microbial metabolites, immune cells, tryptophan metabolism, neural and endocrine pathway: these mechanisms are important regulators of neurotransmitters such as *γ*-aminobutyric acid (GABA) (2). Neuropsychiatric disorders with a possible gastrointestinal etiology include autism, anorexia nervosa, anxiety, Parkinson’s disease, Alzheimer’s disease, attention deficit hyperactivity disorder (ADHD), schizophrenia, bipolar disorders, alcohol dependence, or migraine pain (1). Several preclinical and clinical studies suggested that alterations of microbiota are associated with neuroinflammation (3, 13). Furthermore, this relationship is well supported by studies that have investigated the effects of probiotics, antibiotics, or even germ-free animals on brain activity and function (3, 14, 15).

This narrative review aims at exploring the current knowledge about the connection between PANDAS and the composition of gut microbiota in children, underling the role of the communication along the microbiota gut-brain axis. For this narrative review, the authors have independently searched via PubMed/MedLine database articles published in the last 15 years (2009–2024), based on the following keywords: nutrition; diet; gut microbiota; Pediatric Autoimmune Neuropsychiatric Disorders Associated with Streptococcal Infection. A narrative synthesis approach was used to summarize the results of the included studies. The main relevant case–control studies available regarding the potential association between PANDAS and gut microbiota are two: the first one analyzed the microbiota composition in 30 children with PANDAS or PANS (2), while the most recent one focused on the determination of oxidative stress markers in 30 children with the same diseases (3).

### PANDAS and gut-microbiota composition

According to the articles collected, evidence suggests that streptococcal infections may alter the composition of gut microbiota in pediatric patients with PANDAS, contributing to a persistent inflammatory state that might indirectly influence brain function and individual behavior. In a case–control study by Quagliariello et al. (2), gut microbiota of 30 patients affected by PANS/PANDAS was analyzed and compared with healthy individuals. In younger PANDAS patients (y-PAN, 4–8 years old), reduced microbial diversity (*α*-diversity) has been observed, with a significant increase in Bacteroidetes such as Bacteroides, Odoribacter, and Oscillospira, and reduction of Firmicutes and TM7 (Saccharibacteria) in comparison to healthy controls. The group of patients who discontinued antibiotic therapy and/or probiotic intake 2 to 4 months prior to the study showed higher levels of Bacteroidaceae, Rikenellaceae, and Odoribacteriaceae (2). Conversely, some Firmicutes families including Turicibacteraceae, Tissierellaceae, Gemellaceae, and Carnobacteriaceae (Bacilli class), Corynebacteriaceae, and Lachnospiraceae were absent.

The alteration of the microbiome is linked to crucial metabolic capacities such as glycan degradation and the production of short-chain fatty acids (SCFAs), known for their beneficial effects including anti-inflammatory properties and support for intestinal barrier integrity. When SCFA production is compromised due to altered microbiota, chronic inflammatory state in the gut may worsen, leading to neurological and behavioral alterations (2, 6). Association between high levels of Anti-Streptolysin O (ASLO) titers and specific genera of bacteria such as Dehalobacterium and Lactobacillus have also been observed in the same study, suggesting a complex interaction between immune response, intestinal microbiota, and neural functions (2). Antibodies produced in response to streptococcal infections may influence the composition of the intestinal microbiota, potentially affecting dopamine receptors and other neural processes crucial for brain function, including those involved in tyrosine metabolism associated with neuronal dysfunctions observed in conditions like Parkinson’s disease (2). Regarding treatment, while antibiotics are essential in treating acute Group A beta-hemolytic Streptococcus infections, their prolonged use raises concerns regarding their promotion of intestinal dysbiosis. Antibiotic therapy in PANDAS patients should therefore balance the need to treat the primary infection with preserving the integrity of the gut microbiota. Complementary strategies, such as the use of biotics may be considered to minimize the negative effects of antibiotics on the intestinal microbiota and potentially improve clinical outcomes (16, 17). Other studies confirm the potential link between gut microbiota and psychiatric symptoms, as highlighted by clinical studies utilizing fecal microbiota transplantation (FMT). Two studies conducted on children with Autism Spectrum Disorder (ASD) and gastrointestinal issues demonstrated significant results (18, 19). In the first study (18), 18 children received multiple sessions of FMT from healthy donors after clearing their own gut microbiota. Eight weeks after the last FMT session, gastrointestinal symptoms decreased by 77%, and ASD symptoms improved by 24%. This improvement was associated with an overall increase in microbiota diversity, including higher levels of Bifidobacterium, Prevotella, and Desulfovibrio genera. Two years after treatment, ASD and gastrointestinal symptoms had decreased by 47 and 58%, respectively, compared to pre-treatment levels. The second study (19), conducted on 40 children with ASD and gastrointestinal symptoms, reported similar findings. After 4 weeks of treatment with donor microbiota, gastrointestinal symptoms decreased by 35%, and ASD symptoms improved by 6%. It was observed that patients who responded positively to treatment showed a significant decrease in the prevalence of *Eubacterium coprostanoligenes* compared to non-responders. Additionally, a study involving adults with irritable bowel syndrome (IBS) without psychiatric diagnosis showed that FMT reduced non-clinical symptoms of depression and obsessive-compulsive symptoms. Although these studies are promising and suggest that FMT may positively influence psychiatric symptoms through changes in the composition of the intestinal microbiota, it is important to note that controlled placebo and double-blind studies are still lacking to confirm these effects and to fully understand the underlying mechanisms (6).

### PANDAS and inflammation

Another interesting aspect of available evidence about children with PANDAS is the detection of elevated serum levels of soluble NOX2 derived peptide (sNOX2-dp) and isoprostanes, indicators of elevated systemic oxidative stress and relevant to the manifestation of neuropsychiatric symptoms of the disease. NOX2 activation has also been associated with other inflammatory neurological conditions, suggesting a potential mechanism through which streptococcal infection could influence the pathogenesis of PANDAS (3). Available data (3) also suggest that the immune response triggered by streptococcal infections may lead to the release of lipopolysaccharides (LPS) by gram-negative bacteria, passing from the gut into the bloodstream and potentially contributing to observed neuroinflammation. The evidence of a correlation between elevated levels of serum LPS, sNOX2-dp, and isoprostanes suggests the presence of a potential mechanism through which LPS could promote oxidative stress and neuroinflammation in the context of PANDAS.

The interaction between gut microbiota, antibiotics, and treatments such as prebiotics plays a significant role in gastrointestinal health and potentially also in psychological disorders. However, further research is needed to fully understand the mechanisms involved and optimize the clinical use of these therapies (20).

## Discussion

**Figure 1:**
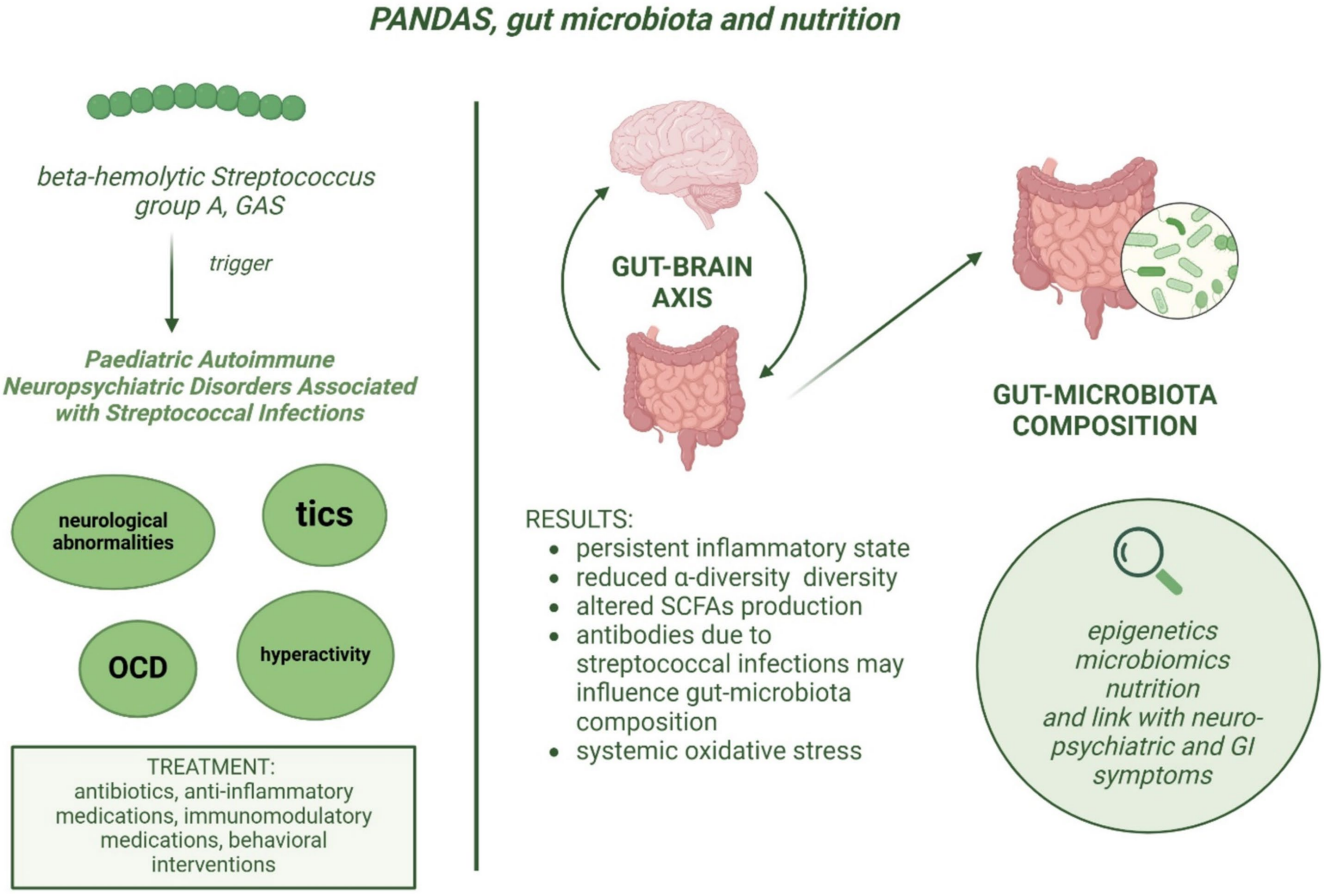
Etiopathogenesis of PANDAS and potential association between dysbiosis and neurological symptoms severity.

The few studies available to date, regarding the gut microbiota composition in children with PANDAS, suggest that this group of patients may have an altered gut microbiota composition compared to healthy controls, as well as a different expression of specific metabolites involved in the inflammatory response, antibody production, and associated with brain function (Figure 1). The study by Loffredo et al. (3) has observed a correlation between increased levels of serum LPS, sNOX2-dp, and isoprostanes, suggesting that LPS might cause oxidative stress and neuroinflammation in PANDAS. Further studies are needed to analyze the association between NOX2 levels and the severity of the neurological manifestations in children with PANDAS, as well as the effects of a healthy dietary pattern useful to promote a healthy gut microbiota, or antioxidant substances on the activity of NOX2 and LPS in this population. The aim of such research would be to obtain new evidence to provide physicians with specific guidelines on managing children with PANDAS in terms of diet and appropriate pharmacological treatment, in order to prevent the worsening of leaky gut and the exacerbation of neurological symptoms.

Increasing data highlight how the severity of symptoms in many neurodegenerative diseases can be linked to gut dysbiosis, thanks to the bidirectional gut-brain communication pathway (21–23). In light of the evidence of gut dysbiosis and the inflammatory state in children with PANDAS, diet could play a role in improving the microbiota composition and thereby reducing the severity of neurological symptoms. Indeed, diet is one of the main factors influencing the composition of gut microbiota and may be responsible for the diversification of the microbial population (24, 25). However, it is important to remember that pediatric patients require an adequate intake of nutrients for proper growth and development, and it is necessary to avoid a restrictive diet that could worsen obsessive-compulsive symptoms and promote the development of eating behavior disorders. As for patients with ASD, the restrictive diet that many patients follow presents challenges in assessing the composition of the gut microbiota, adding complexity in the definition of a nutritional intervention as well. Moreover, considering the impact of antibiotics and of the restrictive diets followed by children, the intervention should be aimed at restoring the correct composition of the gut microbiota. The use of antibiotics in patients with GAS infection may also represent a significant bias in the assessment of gut microbiota composition. Indeed, it is well known that antibiotic treatment may lead to significant alterations, including reduced species diversity, altered metabolic activity and selection of antibiotic-resistant organisms, which can cause antibiotic-associated diarrhea and recurrent *Clostridioides difficile* infections (26). It could be useful to provide guidance on following an anti-inflammatory dietary pattern, such as the Mediterranean diet, while avoiding a Western diet that is high in trans fatty acids, food additives, and ultra-processed foods (27), while ensuring an adequate intake of fibers. A targeted nutritional assessment is essential to identify individual dietary needs and habits, allowing for a personalized intervention plan that addresses each child’s unique health requirements. Despite these considerations, there is currently no evidence supporting a specific diet for managing PANDAS. Therefore, following a Mediterranean diet—known for its anti-inflammatory benefits and balanced nutrient profile (28)—may be advisable, always considering the individual preferences of the children and implementing progressive and personalized interventions based on the Mediterranean diet. To improve the gut microbiota composition through food consumption, it would be advisable to recommend foods rich in fiber (29) but given the selectivity of these patients, promoting a change in dietary habits may be challenging. In addition to foods, the use of prebiotics would have an impact on the microbiota. However, postbiotics could also be considered for their immunomodulatory effects on the immune system (30).

In the pediatric population with PANDAS, a first retrospectively nutrients or food-based dietary pattern analysis could be carried out in order to understand the relationship between diet and health. Further clinical studies could be conducted to evaluate the association between gut microbiota composition and diet.

One of the limitations of the available studies is that both PANDAS and PANS were included in the analysis, providing a heterogeneous case group in terms of etiopathogenesis. Available data in literature are not sufficient to demonstrate whether a different infection than GAS may affect microbiota composition differently, however, a study conducted on murine model revealed that the gut flora presented specific changes according to different antibiotics use and treatment timing (31). Therefore, further studies including a more conspicuous group of selected PANDAS patients are needed to confirm reported data.

A close collaboration is essential among the numerous professionals involved in the pathogenesis and treatment of this complex pathological condition, including pediatricians, rheumatologists, immunologists, neuropsychiatrists, infectiologists, and nutrition experts. A multidisciplinary approach would allow a faster integration of new discoveries, aiming to improve treatment and care strategies. Additionally, it is important to widely disseminate knowledge of these results to general pediatricians, who are the first to manage these patients and can provide an initial effective intervention to modulate the inflammatory response through proper treatment and the implementation of an anti-inflammatory diet.

According to the limited data available, it may be postulated that a relationship between PANDAS and altered gut microbiota composition and that this may contribute to the severity of neuropsychiatric symptoms in these patients. Current treatments, including antibiotics and immunotherapies, are essential but may disrupt gut microbiota, necessitating careful management. Incorporating dietary strategies, aiming to increase the fiber intake could offer beneficial outcomes. A multidisciplinary approach is crucial for integrating new findings into clinical practice and optimizing treatment strategies to improve patient care and outcomes.
